# A synthetic pathogen mimetic molecule induces a highly amplified synergistic immune response *via* activation of multiple signaling pathways†

**DOI:** 10.1039/d1sc00964h

**Published:** 2021-04-01

**Authors:** Naorem Nihesh, Saikat Manna, Bradley Studnitzer, Jingjing Shen, Aaron P. Esser-Kahn

**Affiliations:** Pritzker School of Molecular Engineering, University of Chicago 5801 S Ellis Ave Chicago IL 60637 USA aesserkahn@uchicago.edu

## Abstract

The current understanding of how the immune system processes complex information during natural infections is yet to be exploited for the molecular design of potent immune activators. Here, we address this challenge by design of a pathogen-mimetic molecule that simultaneously co-activates cell-surface active, endosomal and cytosolic immune receptors.

## Abstract 2

Different pathogen components were formulated as a single pathogen mimetic molecule. This combinatorial approach enables us to develop a potent vaccine adjuvant.

## Graphical abstract

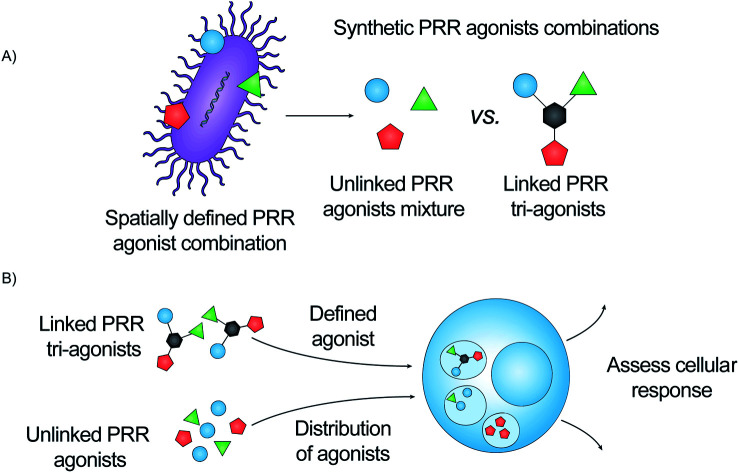

The immune system has evolved to identify complex combinations of microbial patterns and exploit the interdependencies of various microbial signals to generate an effective response during infections.^1^ Whole cell vaccines derived from live pathogens can mimic such responses, thereby generating robust lifelong immunity.^2,3^ However, the generation of a robust response has remained elusive with subunit vaccines that consist of protein antigens. A major roadblock is the rational design of efficacious immunostimulants, or adjuvants employed in these vaccines. For instance, traditionally employed aluminum salt adjuvants primarily induce T-helper cell 2 (Th2) biased responses and fail to generate robust antigen-specific cellular responses.^4–7^ Furthermore, adjuvants developed by targeting pathogen-sensing pathways such as MPLA and CpG, only activate a single immune signaling pathway, thereby poorly mimicking the mechanisms of pathogen recognition. Thus, the design and development of molecules that integrate complex mechanisms of pathogen signaling, to generate robust immune activation still remains both a challenge and necessity.

In our attempt to explore pathogen sensing through molecular design, we previously demonstrated the development of multi-Toll-like receptor (TLR) agonist adjuvants by covalently linking three TLR agonists.^8–10^ These studies employed the use of a triazine scaffold to install unique combinations of three TLR-agonists using sequential orthogonal conjugation chemistry. The TLR tri-agonists synthesized include TLR2/6_4_7, TLR2/6_4_9, TLR1/2_4_9, TLR1/2_4_7, and TLR4_7_9.^8,9^ Each tri-agonist combination elicited unique cytokine responses and generated tailored immune response when applied in vaccines.^9,10^ However, recent studies of various immune signaling pathways during infections have demonstrated the critical importance of crosstalk between different classes of pathogen sensing pathways involving various cellular compartments.^11–15^ For instance, a study by Mellman and coworkers demonstrated that bacterial sensing by cell-surface TLR receptors in innate immune cells leads to the overexpression of endo-lysosomal peptide transporters, and the generation of endosomal membrane tubules. This serves to prime cells for any subsequent detection of bacterial NOD2 ligands at the endosomal membrane.^16^ Furthermore, studies on NLRP3 inflammasome activation – which plays a critical role in pathogen clearance – has demonstrated that a TLR dependent priming signal is necessary for robust NLRP3 activation. This indicates the interdependencies of these two signaling responses.^17–21^

However, such mechanistic findings are yet to be exploited in the design of molecular systems that induce co-operativity and synergism between multiple pathogen-sensing pathways to generate robust immune responses. Hence, the critical question is: can well-known pairwise synergistic immune activation be effectively combined in a synthetic system to generate higher order interactions similar to microbial sensing? To understand the dynamics of such cellular signaling processes at a molecular level, here we report a new class of immunomodulators that elicits activation of three distinct classes of pathogen-sensing pathways involving distinct cellular compartments similar to a pathogen.

The design of our pathogen-mimetic immunomodulator incorporated a surface-active TLR2, an endosomal-membrane active NOD2 and cytosolic NLRP3 inflammasome activating ligands. We thereby assembled Pam_2_CSK_4_ (TLR2/6 agonist, synthetic analogue of bacterial lipoprotein),^22^ Muramyl dipeptide (MDP, NOD2 agonist: the minimal immunomodulatory structure of bacterial cell wall peptidoglycan)^23^ and TAT-GWWWG peptide (cell penetrating peptide, NLRP3 activator)^24^ on a trimeric scaffold (Fig. 1).

**Fig. 1:**
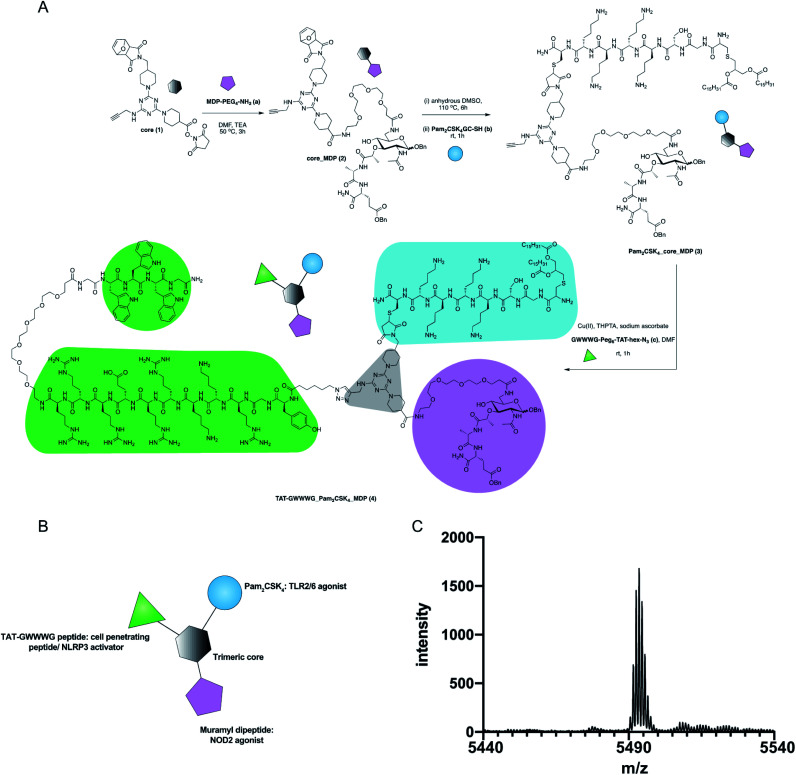
Synthesis of TAT-GWWWG_Pam_2_CSK_4__MDP tri-agonist (4). (A) Synthesis scheme (B) schematic presentation of the linked tri-agonist. (C) MALDI trace of the linked tri-agonist.

The motivation behind this design was to induce crosstalk between each component and enhance NOD2 and NLRP3 inflammasome activation following initial cellular priming by cell-surface TLR2 stimulation.^16,24^ Additionally, we hypothesized that MDP when chemically conjugated to TAT-GWWWG would result in enhanced NOD2 activation and this proved to be true (Fig. 2C). We conjecture that this enhancement in response is the result of increased cytosolic delivery of MDP mediated by TAT-GWWWG.^25–27^

**Fig. 2:**
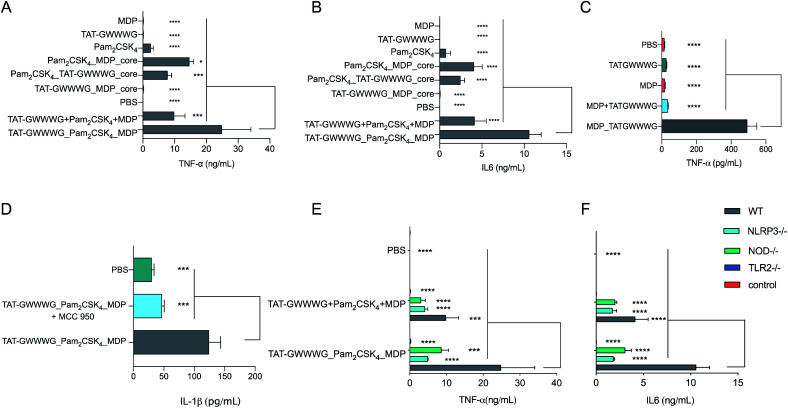
*In vitro* cytokine expression from BMDCs. (A) and (B) Cells were incubated with linked PRR tri-agonist, a 1 : 1 : 1 (molar ratio) mixture of unlinked PRR agonists, various linked di-agonist combinations and single agonists (100 nM each) at 37 °C for 6 h. TNF-α (A) and IL-6 (B) measured by CBA. (C) *In vitro* TNF-α expression from BMDCs as measured by ELISA. Cells were incubated with MPD_TATGWWWG (25 μM) or a 1 : 1 (molar ratio) mixture of the analogous unlinked agonists for 24 h at 37 °C. (D): Analysis of NLRP3 activity (IL-1β secretion) by ELISA. Cells were preincubated with NLRP3 inhibitor MCC-950 (10 mM) for 1 h and then stimulated with linked PRR tri-agonist (10 μM) at 37 °C for 24 h. Inhibition of NLRP3 *via* MCC-950 results in loss of IL-1β activity. (E) and (F) Analysis of TNF-α and IL-6 with WT, TLR2–/–, NOD2–/– and NLRP3–/– cells treated with linked PRR tri-agonist or unlinked agonists (100 nM). Samples were run in triplicate, where *p < 0.05, ***p < 0.001, ****p < 0.0001. Statistical analysis performed using ANOVA with Turkey's multiple comparison test.

The synthesis of our tri-agonist molecule (4) employed sequential conjugation chemistry used previously in the synthesis of TLR tri-agonists.^8,9^ The synthesis started with development of a triazine core (1) with three orthogonal conjugation handles consisting of an NHS ester, an alkyne, and a protected maleimide (ESI, Scheme S1†).^8,9,28^ Following this, amine-functionalized MDP (a, ESI Scheme S2†)^29^ was conjugated to core (1) using NHS chemistry to give core_MDP (2). The core_MDP was then heated to 110 °C for 6 h to reveal active maleimide *via* furan deprotection, following which cysteine-modified Pam_2_CSK_4_ (b) was conjugated *via* thiol–maleimide reaction to give Pam_2_CSK_4__core_MDP (3). This was followed by conjugation of azide-functionalized TAT-GWWWG peptide (c) to Pam_2_CSK_4__core_MDP (3) *via* Cu(i) catalyzed cycloaddition chemistry to afford the final tri-agonist molecule (4, ESI† for synthetic details, purification & characterization). Additionally, all of the dimeric agonist compounds, PAM_2_CSK_4__MDP (3, ESI Scheme S3†), PAM_2_CSK_4__TAT-GWWWG (7, ESI Scheme S4†) and MDP_TAT-GWWWG (8, ESI, Scheme S5†) were also synthesized to measure the contribution of di-agonist components to the immune response.

Following synthesis, we next proceeded to analyze the immunological properties of the tri-agonist. We analyzed the cytokine profile elicited by murine bone marrow-derived dendritic cells (BMDCs) *in vitro* on stimulation with the tri-agonist (100 nM) or equivalent amount of unlinked agonists mixture for 6 h. Parallel studies were performed by incubating equivalent quantities of linked di-agonists and single agonists as well. Analysis of levels of cytokines secreted by agonist stimulation indicated that the linked tri-agonist combination elicited 70% ± 60% (95% confidence interval, CI) higher TNF-α and 160% ± 53% higher IL-6 production compared to the most potent di-agonist combination (Fig. 2A and B). Most notably, compared to an equivalent mixture of unlinked agonists, linking the three ligands boosted the synergistic IL-6 secretion by 155% ± 52% and TNF-α secretion by 151% ± 95% (Fig. 2A and B). These results indicated that synergistic cellular co-activation by localization of these microbial signals induced crosstalk to enhance immune activation.^30^ With these exciting results, we next proceeded to analyze whether the tri-agonist activated all the target pathogen sensing pathways. We thereby analyzed cytokine secretion elicited by the tri-agonist stimulation on wild type (WT) BMDCs along with TLR2, NOD2 and NLRP3 knockout BMDCs (Fig. 2E and F). It was observed that TLR2 knockout BMDCs expressed near background level cytokines indicating that TLR signaling orchestrates the immune activation and primes other signaling pathways. Intriguingly, the NOD2 knockout BMDCs expressed 65% ± 54% lower TNF-α and 70% ± 20% lower IL-6 levels compared to the WT BMDCs implying a significant enhancement in immune response due to crosstalk between NOD2 and TLR2 receptors. Similarly, NLRP3 knockout BMDCs elicited lower cytokine responses (TNF-α, IL-6) compared to WT BMDCs (Fig. 2E and F). However, such effects might also be the result of reduction in MDP activity observed in NLRP3–/– cells. To further validate NLRP3-inflammasome activation by the tri-agonist, we analyzed for secretion of IL-1β, a common marker of inflammasome activation.^19,21^ Stimulation with the tri-agonist resulted in 600% ± 130% higher levels of IL-1β compared to an equivalent mixture of unlinked agonists (ESI, Fig. S1†). Additionally, studies with the tri-agonist with WT BMDCs along with MCC-950, a NLRP3 specific inhibitor displayed a 62% ± 20% reduction in IL-1β activity, thereby validating NLRP3 activation (Fig. 2D).^31,32^

Our studies indicated complex dynamics of cellular co-activation. To further analyze these responses, we studied the transcriptional response in BMDCs upon stimulation with unlinked agonists and linked tri-agonist. The RNA of BMDCs was extracted after 6 h of stimulation with the agonists or PBS and differential gene expression was compared. As seen in the heatmap (Fig. 3A), we observed a similar trend in responses when the BMDCs were activated with the unlinked agonists or linked tri-agonist.

**Fig. 3:**
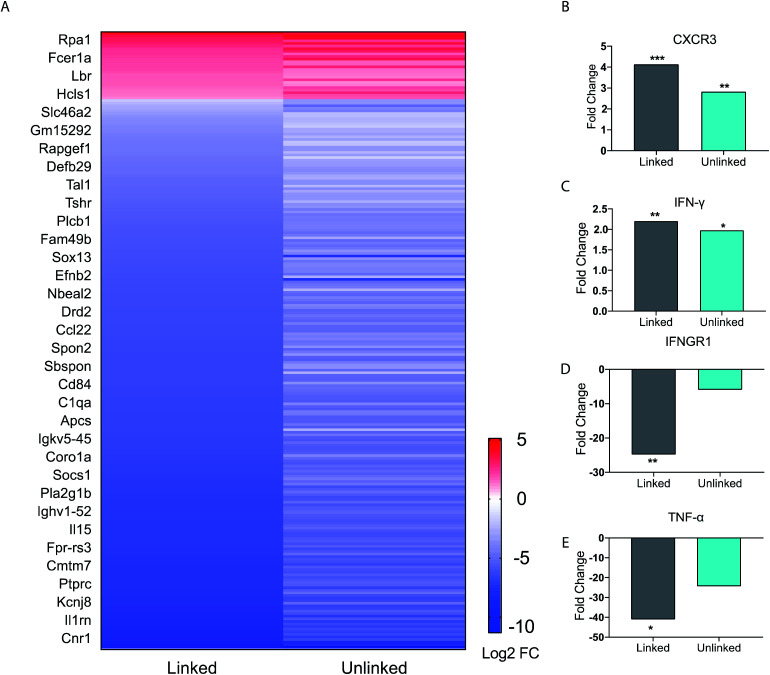
BMDC gene expression profile data. (A) Heat map of immune function related genes. Each figure represents the average of three independent experiments. BMDCs were incubated as untreated, or with either the linked or unlinked tri-agonist combination for 6 h at 37 °C. RNA was then extracted and sequenced on a NextSeq550. The gene expression of the BMDCs in response to unlinked and linked tri-agonist stimulation was compared to unstimulated BMDCs to determine the differential gene expression profiles. Included in the heatmap are only immune-associated genes with *p* value <0.05 for either the linked or unlinked tri-agonists relative to unstimulated BMDCs and a 2-fold change in expression. (B)–(E) Fold change in gene expression for CXCR3, IFN-γ, IFNGR1, TNF-α, in BMDCs in response to linked and unlinked tri-agonist combination where *p < 0.05, **p < 0.01, and ***p < 0.001. *P*-Values are calculated relative to unstimulated BMDCs.

However, key differences exist in magnitude of this regulation. For instance, compared to the unlinked mixture, the linked tri-agonist generated higher expression of CXCR3, a key receptor for interferon induced chemo-attractants that helps differentiate naïve T cells into T_h_1 effector T cells (Fig. 3B).^33,34^ Additionally, it induced greater differences in expression for both interferon-gamma (IFN-γ) and interferon gamma receptor 1 (IFNGR1) – which dimerizes with IFNGR2 to detect IFN-γ (Fig. 3C and D). The greater upregulation of IFN-γ, along with the greater downregulation of IFNGR1, indicates the tri-agonist induces high levels of IFN-γ signaling. This can possibly be an effect of enhanced IL-18 secretion due to inflammasome activation by the linked tri-agonist.^35^ We hypothesize this high expression of CXCR3 and IFN-γ with the linked tri-agonist would induce robust tailored T cell responses, specifically T_h_1 polarized responses.

Although high cytokine levels of TNF-α was observed at 6 h, the transcriptional levels of TNF-α were strongly downregulated at the same time point. This difference in response is likely down to kinetics, as TNF-α is an inflammatory response gene that does not require new protein synthesis (Fig. 3E). As a result, TNF-α transcription is commonly upregulated at earlier timepoints and peaks within 2 h of stimulation, supporting this difference in the transcriptional levels compared to the cytokine levels.^36^

With promising *in vitro* results and the indication of robust tailored cellular responses, we proceeded to determine if amplified immune response from the linked tri-agonist would translate to its use as a vaccine adjuvant *in vivo*. Groups of five mice were immunized *via* intramuscular (IM) injection with ovalbumin (OVA, 100 mg) adjuvanted with linked tri-agonist (5 nmol) or equivalent quantities of unlinked agonist mixture. Vaccines were formulated in Addavax (AV), an MF-59 like oil-in-water nano-emulsion.^37^ Parallel studies were also performed with unadjuvanted OVA or OVA admixed with Addavax as controls. Mice were boosted in an identical fashion on day 14. On day 24 blood sera were collected to analyze for antibody titer (Fig. 4A) and splenocytes were harvested to analyze for antigen-specific T cell responses (Fig. 4B and C). It was observed that the linked tri-agonist formulation enhanced IFN-γ secreting CD4^+^ T cells response by 200% ± 60% and IFN-γ secreting CD8^+^ T cells response by 40% ± 30% compared to unlinked combination of agonists (Fig. 4B and C). It also elicited 300% ± 115% higher antibody responses compared to OVA/Addavax formulation (Fig. 4A). However, the antibody response to the linked and the unlinked combination was similar. There can be various reasons for this observation. Mainly, just as TLR2 activation was the main driver of the *in vitro* cytokine response (Fig. 2E and F), it is possible that the antibody response is also primarily driven by TLR2 engagement. As a result of which there is not much difference between the linked and the unlinked combinations. Moreover, the most important find is that the linked combination enhances both CD8^+^ and CD4^+^ T cell response relative to the unlinked combination. It can be envisioned that the adjuvanticity of the tri-agonist can be improved by making structural changes to the construct. We are currently exploring the use of D configuration amino acids instead of L amino acids for the synthesis of TAT-GWWWG which will possibly make it more resistant to proteolysis. Also, the potency of the tri-agonist maybe improved by using Murabutide, a more potent NOD2 activator compared to Muramyl dipeptide. These changes will likely improve the NOD2 and NLRP3 activation and improve humoral and cellular responses. Additionally, formulation development to enhance pharmacokinetics may improve the activity of the tri-agonist.

**Fig. 4:**
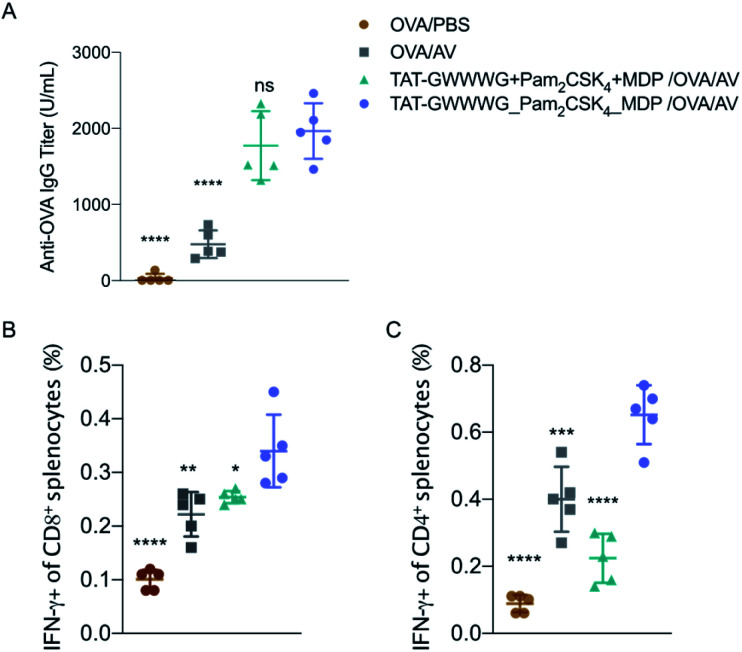
*In vivo* vaccination studies. Mice (*n* = 5) were vaccinated on day 0 with OVA (100 μg) adjuvanted with PBS (vehicle control), or Addavax (25 μL), or 5 nmole each of unconjugated multi-PRR agonist, in Addavax (AV, 25 μL), or 5 nmole of linked tri-agonist in Addavax (25 μL). Final volume of each formulation was made 50 μL with PBS. Mice were given a vaccine boost on day 14. On day 24, sera, spleens were collected from mice. (A) Antibody titer as measured by ELISA. (B) and (C) T cell response as measured by intracellular cytokine staining. ns = non-significant, *p < 0.05, **p < 0.01, ***p < 0.001, ****p < 0.0001. Statistical analysis is performed between the linked tri-agonist and indicated groups using ANOVA by the Turkey's multiple comparison test.

Nevertheless, these results demonstrated that the tri-agonist served as a potent adjuvant *in vivo* which provides a unique immune stimulatory method with the potential to improve cellular responses in vaccine adjuvants.

## Conclusions

In our previous work on polyvalent TLR agonist we demonstrated that TLR tri-agonist stimulate distinct, combination-dependent innate immune responses. Building on the understanding on dynamics of cellular co-activation of innate immune signaling pathways, here we developed a small-molecule tri-agonist inspired by the mechanisms of stimulation of multiple different pattern-sensing pathways by a pathogen. It gave us insight into how pathogen-sensing pathways respond to multiple input signals as is often the case with infections. Our data reveal that the complex combinatorial logic of pathogen sensing pathways can be incorporated in molecular design to modulate the innate immune system. Our studies also indicated that trimeric combinations of pairwise interactions between multiple pathogen sensing pathways can generate complex higher-order interactions that is not achieved in an unconstrained system or with dimeric interactions. This work thus provides a framework to harness complex pathogen sensing in designing efficacious pathogen-mimetic immune therapies. This study thus provides a framework to rationally design other multi-agonist constructs with highly synergistic immune activation. We are currently expanding our toolkit and employing the tri-agonists for application in various infectious disease vaccines.

## Author contributions

NN and SM conceived the research and performed experiments. JS assisted with *in vivo* experiment and BS performed RNA sequencing and analysis. NN and SM wrote the manuscript. AEK supervised the research. All authors edited the manuscript.

## Conflicts of interest

There are no conflicts to declare.

## Supplementary Material

SC-012-D1SC00964H-s001
