# Supplementary material for: A synthetic pathogen mimetic molecule induces a highly amplified synergistic immune response via activation of multiple signaling pathways
Source: Chem Sci. 2021 Apr 1;12(19):6646–51. doi: 10.1039/d1sc00964h (PMC8132936; doi:10.1039/d1sc00964h)
Supplement: SC-012-D1SC00964H-s001 [file SC-012-D1SC00964H-s001.pdf]

Electronic Supplementary Material (ESI) for Chemical Science.
This journal is © The Royal Society of Chemistry 2021

Supporting Information

A Synthetic Pathogen Mimetic Molecule Induces a Highly Amplified Synergistic Immune Response via Activation of Multiple Signaling Pathways

Naorem Nihesh, ‡[a] Saikat Manna, ‡[a] Bradley Studnitzer[a], Jingjing Shen[a], Aaron P. Esser-Kahn. *

Materials and Methods

Reagents and Instrumentation. Unless otherwise stated all chemicals were purchased from commercial sources and were used as received. All chemical reactions were carried out in dried glassware using anhydrous solvents under Argon gas. Reagents were purchased from Sigma-Aldrich, Bachem, ThermoFisher, Quanta Biodesign, Anaspec, Broadpharm or Acros Organics. Buffers and media for cell culture were purchased from Fisher Life Technologies. Addavax was purchased from Invivogen. Absorbance for ELISA was measured on a Fisher Scientific MultiSkan FC. Cytometric bead array data was acquired on a Novocyte flow cytometer. ^{1}H and ^{13}C NMR spectra were taken on a Bruker 500 NMR spectrometer (500 MHz) or 400 NMR spectrometer (400 MHz) and analyzed using MestreNova software. Spectra are referenced to solvent peak for 1H NMR (CD3OD = 3.33 ppm, (CD3)2SO = 2.50 ppm, CDCl3 = 7.26 ppm) and 13C NMR (CD3OD = 49.00 ppm, (CD3)2SO = 39.52 ppm, CDCl3 = 77.16 ppm). Peptides were synthesized via solid phase peptide synthesis on an automated microwave peptide synthesizer (Liberty Bllue, CEM) Analytical high-performance liquid chromatography (HPLC) was performed using an Agilent 1260 Infinity HPLC with a Phenomenex Luna 3 µm C8 100 Å 150 x 4.6 mm LC column. Preparative HPLC was performed on a Gilson Preperative HPLC System with 333 HPLC Pumps and GX-271 liquid handler using a Phenomenex Luna 5 µm C8(2) 100 Å 150 x 21.2 mm LC column. Electrospray ionization – mass spectrometry (ESI-MS) was performed on an Agilent 6130

LC MS. MALDI-TOF was performed on Bruker ultraflextreme MALDI-TOF-TOF system. Silica Gel Chromatography was performed using RediSep Rf normal silica columns on a Teledyne-Isco CombiFlash Rf auto column instrument. Data was analyzed using one-way ANOVA in Graph Pad Prisim software. All values were reported as mean ± SD, where error bars represent biological replicates.

BMDC activation studies: Bone marrow derived dendritic cells (BMDCs) were harvested from the femurs of 6-week-old C57BL/6 mice (Jackson Laboratory). BMDCs were cultured in BMDC primary medium: RPMI 1640 (Life Technologies), 10% heat inactivated fetal bovine serum (FBS), 20 ng/mL granulocyte-macrophage colony-stimulating factor (GM-CSF), 2 mM L-glutamine (Life Technologies), antibioticantimycotic (1×) (Life Technologies), and 50 μM betamercaptoethanol (all components were 0.2 μM sterile filtered together before use). For BMDC activation studies, 2×10^5 cells per well were seeded in round-bottom 48-well plates and treated with various concentrations of linked PRR tri-agonist or 1:1:1(molar ratio) mixture of the analogous unlinked PRR agonists or various linked di-agonist combination, then incubated at 37 °C. The supernatants were collected after 6 h or 24 h. Cytokine concentration in the media were measured either by mouse inflammation kit CBA (BD Biosciences) or ELISA kits (BioLegend) following manufacturer's instructions.

NLRP3 inhibition studies

For NLRP3 inhibition studies, 2×10^5 cells per well were seeded in round-bottom 48-well plates and treated with 10 μM of MCC-950 at 37 ^{o}C for 1 h followed by addition of 10 μM of PRR tri-

agonist for 24 h. Samples were collected and analyzed for IL-1b by ELISA kit (BioLegend) as per manufacturer's instructions.

***In-vitro* cytokine analysis by cytometric bead array (CBA).** CBA mouse inflammation kit was purchased from BD Biosciences. BMDCs were plated in 48 well plates (4 x 10^5 cells in 400 µL) and stimulated with indicated sample for 6 h. The supernatant was transferred to Eppendorf tubes and centrifuged at 1000 x g for 10 minutes. The supernatant was removed and diluted by a factor of 2.5. The assay was then performed following manufacturers protocol and analyzed by a Novocyte flow cytometer. Data were analyzed using Graphpad Prism software.

***In-vitro* cytokine analysis by ELISA.** BMDCs were plated in 48 well plates (4 x 10^5 cells in 400 µL) and stimulated with indicated sample at 37 ^{o}C for 24 h. The supernatants were assessed by ELISA for IL-1b. ELISA kits were purchased from Biolegend and used according to instruction manual. Samples were undiluted.

RNA sequencing and analysis. BMDCs were incubated with the linked and unlinked tri-agonist combinations for 6 h. RNA was extracted using a Direct-zol RNA-Microprep kit (Zymo), prepped using SMARTer® Stranded Total RNA-Seq Kit v2 (Takara), and sequenced on a NextSeq550 (Illumina). RNA seq reads were mapped to GRCm38 mouse reference genome using STAR version 2.7.0b. The resulting files from the alignment step above were taken to evaluate transcriptional expression using subread::featureCounts with gencode transcript annotation M19 by comparing BMDc's with linked or unlinked trimer activation to unstimulated BMDCs. The obtained count table was normalized and log fold change in expression was generated using the

edgeR package. Immune genes with pval <.05 and 2-fold differential expression for at least one of the dosing conditions are considered in the analysis.

***In-vivo* studies.** All animal experiments were approved by Institutional Animal Care and Use Committee (IACUC), University of Chicago (72517). Mice (n=5) were vaccinated on day 0 with OVA (100 ug) adjuvanted with PBS (vehicle control), or Addavax (25 uL), or 5 nmole each of unconjugated multi-PRR agonist (TAT-GWWWG+Pam_2CSK_4+MDP) in Addavax (AV, 25 uL), or 5 nmole of conjugated agonists (TAT-GWWWG_Pam_2CSK_4_MDP) in Addavax (25 uL). Final volume of each formulation was made 50 uL with PBS. Mice were given a vaccine boost on day 14. On day 24, sera and spleens were collected from mice. Antibody titer was measured by ELISA and T cell response was measured by intracellular cytokine staining.

T-cell recall assays: Spleens were collected from mice on day 24 and incubated in ice-cold RPMI until processing. Spleens were processed into a single-cell suspension via mechanical disruption and passaged through a 70 μm strainer. The splenocytes were washed with PBS and then treated with RBC lysis buffer for 3 min at room temperature. The single-cell suspension was washed with PBS and resuspended in RPMI. These single cell suspensions were then plated at a density of 10^7 cells/mL and treated with respective peptide epitopes (20 mg/mL). Following two hours of incubation, golgi plug (Brefeldin A) was added and the cells were additionally stimulated for 6 h more. Following incubation, cells were stained with viability stain and for appropriate cell surface markers (CD4, CD8) and intracellular cytokine staining was performed for IFN-γ. Samples were analyzed on a NovoCyte 3000 flow using the NovoExpress software. Total numbers of spleen/LN

lymphocytes were back-calculated from the number of marker-positive cells read and the total volume of sample processed by the NovoCyte 3000 flow cytometer.

Measurement of the anti-OVA IgG response: Blood was collected by cardiac puncture from mice on day 24 and serum was separated by centrifugation and stored at −20 °C. Sera was assayed for antibody levels against OVA using ELISA kit (Alpha Diagnostics) following manufacturer's protocol. The absorbance was measured at 450 nm in a microtiter-plate spectrophotometer using a blank measurement at 620 nm.

Results and Discussion

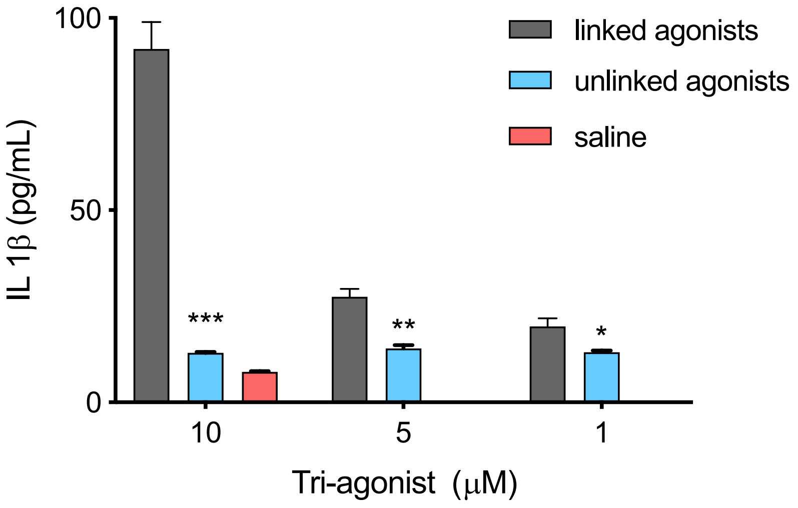

Figure S1: In vitro cytokine expression from BMDCs as measured by ELISA (IL-1β). Cells were incubated with PRR triagonist (100 nM, 1 μM or 10 μM) or a 1:1:1 (molar ratio) mixture of the

analogous unlinked TLR agonists for 24 h at 37 °C. Error bars represent standard deviation of the mean. Samples were run in triplicate, where * $p < 0.05$; ** $p < 0.01$, *** $p < 0.001$. Statistical analysis is between the linked tri-agonist and the unlinked mixture, performed using ANOVA by the Turkey's multiple comparison test.

Synthetic schemes and procedures

Peptide synthesis: Peptides were synthesized on an automated microwave peptide synthesizer by Fmoc solid phase peptide synthesis. A low-loading Rink Amide resin (Sigma Aldrich, 0.34 meqg^{-1}) was used for all peptide synthesis. Peptide couplings were performed using DIC, Oxyma at 90 ^{o}C for 3 minutes per coupling except for the coupling of Arginine, in which case the coupling reaction went up to 10 minutes at 90 ^{o}C for two cycles. The Fmoc group was deprotected with 20 % piperidine in DMF (v/v) at 75 ^{o}C for 5 minutes. Azido hexanoic acid was used to modify the N terminus of the TAT-GWWWG peptide. A PEG_6 spacer was installed between the TAT peptide and the GWWWG sequence. Peptides were cleaved in 95% trifluoroacetic acid, 2.5% H2O and 2.5% triisopropylsilane, for 4 h at 25 °C. After precipitation in ice-cold diethyl ether, peptides were dried, resuspended in 0.1 % TFA in 50:50 water/acetonitrile mixture and purified by HPLC on a C8 or C18 column using a gradient of 0.1% TFA in acetonitrile. Peptide masses were confirmed by matrix-assisted laser desorption/ionization–time of flight mass spectrometry (MALDI-TOF).

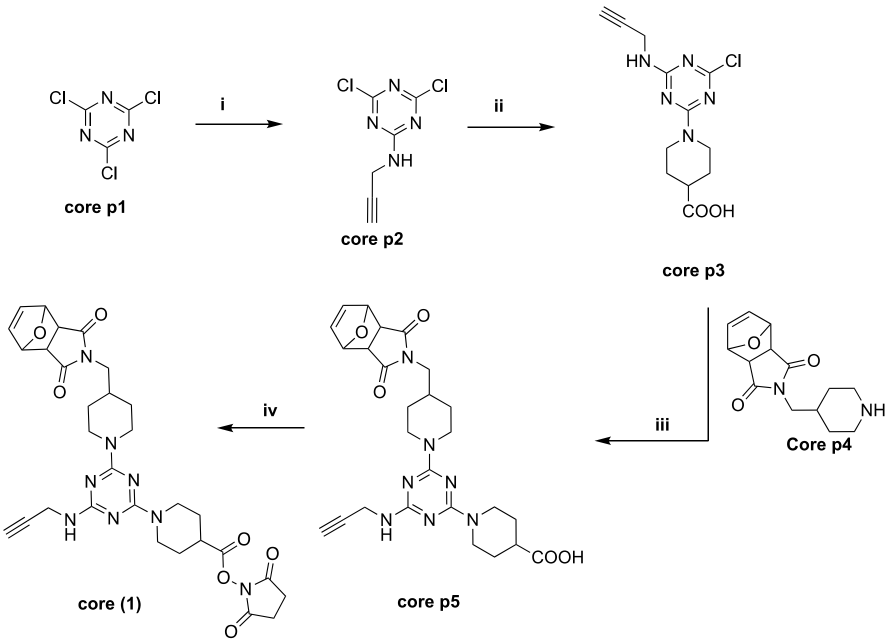

Scheme S1: Synthetic route of **core (1)**. Reagents and conditions: (i) propargyl amine, DIPEA, THF, 0 ^oC, 3 h 80%; (ii) isonipecotic acid, DIPEA, H_2O: DMF (20:80), 80 ^oC, 12 h, 70%; (iii) DIPEA, DMF, 60 ^oC, 6 h, 50%; (iv) N'-Dissuccinimidyl carbonate, DMAP, DMF, rt, 6 h, 87%.

core p3: core p2[1] (200 mg, 1 mmol) was dissolved in DMF (10 mL). DIPEA (200 uL, 1.1 mmol) and isonipecotic acid (150 mg, 1.15 mmol) were added to the solution. The mixture was heated at 80 ^oC for 12 h. The reaction was monitored by mass spectrometry. The reaction was then concentrated and purified by column chromatography (1% MeOH/ EtOAc with 1% NH_4OH). The product was a white powder (170 mg, 70%). ^1H NMR (400 MHz, DMSO-d_6) δ 12.47 (s, 1H), 8.37 (s, 1H), 4.68 (d, J = 13.1 Hz, 1H), 4.53 (d, J = 13.0 Hz, 1H), 4.18 (s, 2H), 3.35 – 3.16 (m, 3H), 2.73 (m, 1H), 2.05 (m, 2H), 1.63 (m, 2H). ^{13}C NMR (101 MHz, DMSO) δ 175.50, 168.44, 164.99, 163.63, 80.76, 72.90, 53.55, 42.39, 29.72, 27.67, 18.06, 16.72. ESI-MS: m/z calc'd for $C_{12}H_{14}ClN_5O_2$ [M+H]$^+$ 296.3, observed 296.1.

core p5: **core p3** (100 mg, 0.33 mmol), **core p4**[3] (110 mg, 0.4 mmol) and DIPEA (100uL, 0.55 mmol) were dissolved in DMF (5 mL) and heated to 60 °C for 6 h. The reaction was monitored by mass spectrometry. The reaction was then concentrated and purified by column chromatography (1% MeOH/EtOAc with 1% NH_4OH). The product was a white powder (60 mg, 0.11 mmol, 35%). ^1H NMR (400 MHz, DMSO-d_6) δ 12.00 (s, 1H), 7.56 (s, 1H), 6.55 (s, 2H), 5.14 (s, 2H), 4.51 (s, 4H), 4.10 (s, 2H), 3.27 (d, J = 7.2 Hz, 2H), 3.14 – 2.99 (m, 3H), 2.93 (s, 2H), 2.86 (s, 2H), 2.54 (t, J = 4.0 Hz, 1H), 1.96 – 1.80 (m, 3H), 1.61 (d, J = 12.7 Hz, 2H), 1.48 (s, 2H), 1.06 (s, 2H)^{13}C NMR (101 MHz, DMSO) δ 176.76, 175.47, 161.47, 158.90, 158.55, 136.48, 80.44, 73.34, 53.56, 47.13, 43.56, 43.20, 42.88, 41.82, 33.96, 29.86, 28.66, 27.76, 18.04, 16.72. ESI-MS: m/z calc'd for $C_{26}H_{31}N_7O_5$ M+H]$^+$ 522.3, observed 522.2.

core (1): core p5 (20 mg, 0.038 mmol) was dissolve in DMF (5 mL). DMAP (1 mg, 0.007 mmol) and N'-Dissuccinimidyl carbonate (20 mg, 0.08 mmol) were added to the solution. The solution was stirred at rt for 6 h. The reaction was monitored by mass spectrometry. After HPLC purification (Solvent A: 0.1 % TFA in water, solvent B: 0.1% TFA in acetonitrile. Gradient: t 0-20 min, ramp 10% B to 90% B), fractions containing **core** were pooled and concentrated to give 65 % yield. ESI-MS: m/z calc'd for $C_{30}H_{34}N_8O_7$ [M+H]$^+$ 619.2, observed 618.9.

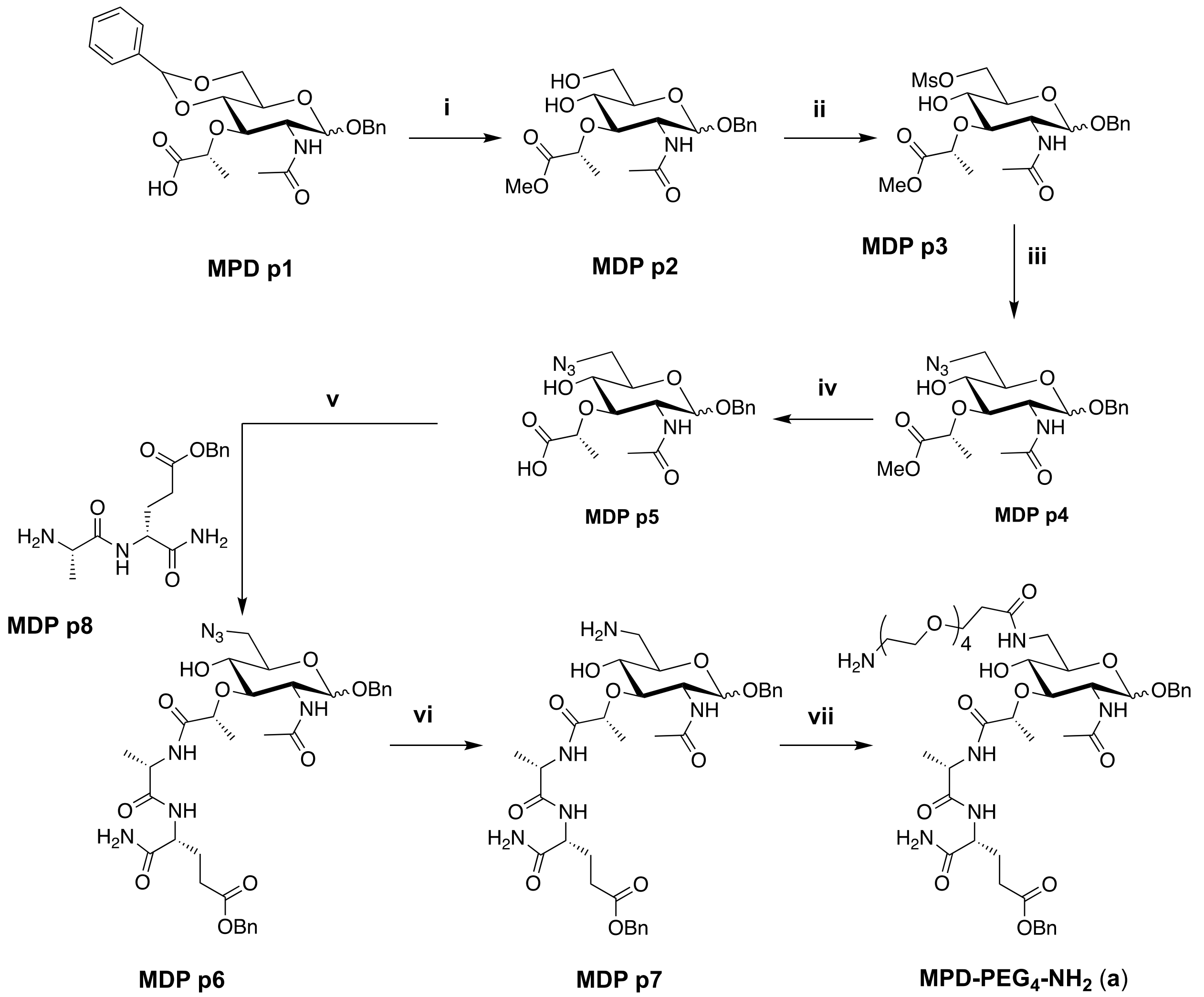

Scheme S2[2]: Synthetic route of **MDP-PEG$_4$-NH$_2$ (a)**. Reagents and conditions: (i) IRA H^+ Resin, MeOH, reflux, 4 h, quantitative; (ii) p-toluene sulfonic acid, methyl sulfonyl chloride, pyridine, -10 ^{o}C, 78% 4 h; (iii) sodium azide, DMF, 70 ^{o}C, 16 h, 77%; (iv) potassium hydroxide; (v) HATU, DIPEA, DMF, rt, 3 h 63%; (vi) TCEP, MeOH, 60 ^{o}C, 5 h, 82%; (vii) (1) azido-PEG$_4$-NHS ester, DIPEA, DMF, 45 ^{o}C 3 h; (2) TCEP, MeOH, 60 ^{o}C, 2 h, 87%.

MDP p7: MDP p6 (25 mg, 0.035 mmol) was dissolved in methanol (10mL) and 1 M TCEP solution in water (2 mL) was added. The solution was heated to 60 ^{o}C for 5 h. The reaction was

monitored by mass spectrometry and was stopped when **MDP p6** could no longer be detected. The solvent was removed, and the residue was dissolved in 0.1% TFA/H_20 (1 mL). **MDP p7** was purified by HPLC (Solvent A: 0.1 % TFA in water, solvent B: 0.1% TFA in acetonitrile. Gradient: t 0-20 min, ramp 10% B to 90% B). Desired fractions were pooled and concentrated to give **MDP p7** (0. 020 g, 0.029 mmol, 82%).

^{1}H NMR (400 MHz, DMSO-d_6) δ 8.14 (d, J = 8.2 Hz, 1H), 8.09 (d, J = 8.2 Hz, 1H), 7.57 (d, J = 6.9 Hz, 1H), 7.40-7.31 (m, 10H), 7.08 (s, 1H), 5.30 (d, J = 6.9 Hz, 2H), 5.07 (s, 2H), 4.74 (d, J = 3.5 Hz, 1H), 4.67 (d, J = 12.5 Hz, 1H), 4.44 (d, J = 12.5 Hz, 1H), 4.27 (m, 1H), 4.19 (m, 1H), 4.10 (m, 1H), 3.81 (m, 1H), 3.66 (m, 1H), 3.57 – 3.45 (m, 3H), 3.17 (d, J = 5.0 Hz, 2H), 2.35 (t, J = 7.9 Hz, 2H), 2.01 (m, 1H), 1.78 (s, 4H), 1.24 (d, J = 6.7 Hz, 3H), 1.21 (d, J = 6.9 Hz, 3H). ^{13}C NMR (101 MHz, DMSO) δ 171.28, 171.02, 170.48, 170.46, 167.89, 136.08, 134.52, 126.78, 126.56, 126.34, 126.24, 125.92, 125.84, 94.29, 77.48, 74.81, 71.58, 67.99, 66.21, 63.84, 60.43, 58.94, 51.27, 49.81, 46.95, 46.54, 28.37, 25.37, 20.96, 17.37, 16.70. ESI-MS: m/z calc'd for $C_{33}H_{45}N_5O_{10}$ $[M+H]^+$ 673.2, observed 673.4.

MDP-PEG$_4$-NH2: **MDP p7 (**20 mg, 0.029 mmol**),** azido-PEG$_4$-NHS ester (22mg, 0.060 mmol) and DIPEA (6 uL, 0.035 mmol) were dissolved in DMF (5 mL) and heated to 45 ^{o}C for 3 h. The reaction was monitored by mass spectrometry and stopped when **MDP p7** could no longer be detected. 1 M TCEP solution in water (2 mL) was added and the temperature was increased to 60 ^{o}C and maintained for 2 h. The reaction was monitored by mass spectrometry. **MDP-PEG$_4$-NH2** was purified by HPLC (Solvent A: 0.1 % TFA in water, solvent B: 0.1% TFA in acetonitrile. Gradient: t 0-20 min, ramp 10% B to 90% B). Fractions containing **MDP-PEG$_4$-NH2** were pooled and concentrated to give a yield of 55%**.**

^{1}H NMR (400 MHz, DMSO-d_6) δ 8.17 (d, J = 8.3 Hz, 1H), 8.10 (d, J = 8.2 Hz, 1H), 8.03 (t, J = 5.7 Hz, 1H), 7.79 (s, 3H), 7.56 (d, J = 7.0 Hz, 1H), 7.38 – 7.30 (m, 10H), 7.09 (d, J = 2.0 Hz, 1H), 5.08 (s, 2H), δ 4.74 (d, J = 3.5 Hz, 1H), 4.64 (d, J = 12.4 Hz, 1H), 4.42 (d, J = 12.4 Hz, 1H), 4.27 (m, 2H), 4.19 (m, 1H), 3.85 (m, 1H), 3.63 – 3.40 (m, 20H), 3.24 – 3.11 (m, 2H), 2.98 (m, 2H), 2.45 – 2.30 (m, 4H), 2.06 – 1.96 (m, 1H), 1.78 (s, 4H), 1.24 (d, J = 6.7 Hz, 3H), 1.21 (d, J = 6.9 Hz, 3H). ^{13}C NMR (101 MHz, DMSO) δ 173.77, 173.08, 172.70, 172.24, 170.99, 169.78, 137.65, 136.24, 128.53, 128.32, 128.10, 127.97, 127.76, 127.67, 95.95, 78.76, 76.58, 70.92, 69.79, 68.04, 66.71, 65.59, 52.99, 51.60, 48.30, 38.72, 35.98, 30.11, 27.06, 22.67, 19.04, 18.42. ESI-MS: m/z calc'd for $C_{44}H_{66}N_6O_{15}$ [M+H]$^+$ 919.4, observed 919.3.

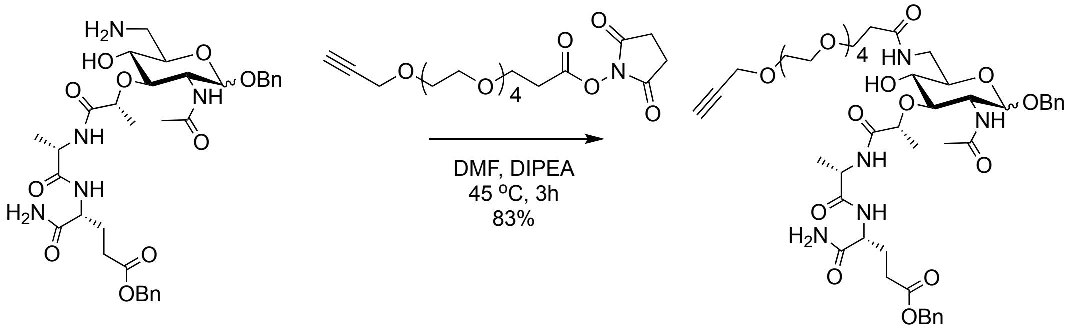

MDP p7

MPD-Peg$_4$-alkyne (d)

MDP-PEG$_4$-alkyne: MDP p7 (20 mg, 0.029 mmol**),** alkyne-PEG$_4$-NHS ester (25 mg, 0.060 mmol) and DIPEA (6 uL, 0.035 mmol) were dissolved in DMF (5 mL) and heated to 45 °C for 3 h. The reaction was monitored by mass spectrometry and stopped when **MDP p7** could no longer be detected. **MDP-PEG$_4$-alkyne** was purified by HPLC (Solvent A: 0.1 % TFA in water, solvent B: 0.1% TFA in acetonitrile. Gradient: t 0-20 min, ramp 10% B to 90% B). Fractions containing **MDP-PEG$_4$-alkyne** were pooled and concentrated to give a yield of 83%. ESI-MS: m/z calc'd for $C_{47}H_{67}N_5O_{16}$ [M+H]$^+$ 958.5, observed 958.3.

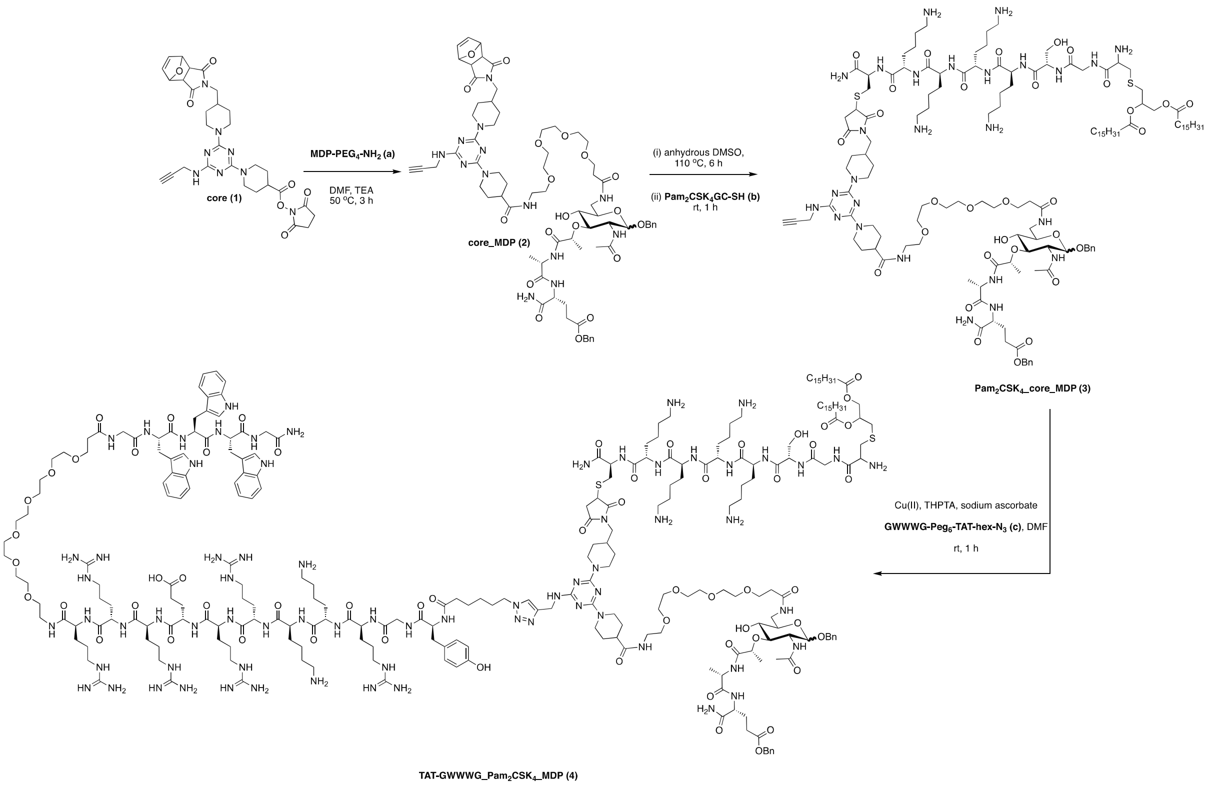

Scheme S3: Synthetic route of **TAT-GWWW_Pam$_2$CSK$_4$_MDP (4)** tri-agonist.

core_MDP (2): MDP-PEG$_4$-NH2 (a) (10 mg, 0.01 mmol)**, core (1)** (9 mg, 0.013 mmol) and TEA (5µL) were dissolved in DMF (5 mL). The mixture was stirred at 50 °C for 3 h. The reaction was monitored by mass spectrometry and was stopped when **MDP-PEG$_4$-NH2 (a)** could no longer be detected. The solvent was removed, and the residue was dissolved in 0.1% TFA/H$_2$0 (1 mL). The compound was purified by HPLC (Solvent A: 0.1 % TFA in water, solvent B: 0.1% TFA in acetonitrile. Gradient: t 0-20 min, ramp 10% B to 90% B). Desired fractions were pooled and concentrated to give **core_MDP (2)**. (11 mg, 0.008 mmol, 77%). ESI-MS: m/z calc'd for $[M+H]^+$ 1422.7 observed 1422.5.

Pam_2CSK_4_core_MDP (3): Furan protected **core_MDP (2)** (10 mg, 0.007 mmol) was dissolved in anhydrous DMSO (3 mL) and stirred for 6 h at 110 °C to expose the maleimide. The reaction was monitored by mass spectrometry. When furan was completely deprotected, the solution was cooled to room temperature. **Pam_2CSK_4GC (b)** (10 mg, 0.007 mmol) and DIPEA (500 uL) were added to the solution and stirred for 1 h. The reaction was monitored by mass spectrometry. **Pam_2CSK_4_core_MDP (3)** was purified by HPLC (Solvent A: 0.1 % TFA in water, solvent B: 0.1% TFA in acetonitrile. Gradient: t 0-20 min, ramp 10% B to 90% B) to give a yield of 52%. ESI-MS: m/z calc'd for $[M+H]^+$ 2784.6 observed 2784.8

TAT-GWWWG_ Pam_2CSK_4_MDP (4): Pam_2CSK_4_core_MDP (3) (5 mg, 0.002 mmol) was dissolved in DMF (200 μL). **GWWWG-peg$_6$-TAT-hex-N_3 (c)** (5 mg, 0.002 mmol) dissolved in DMF (200 μL) were added to the solution. 200 μL of CuSO4•5H_2O (20 mM) solution and 400 μL of THPTA (50 mM) solution were mixed and added to the mixture. 200 μL of sodium ascorbate (100 mM) was then added to the reaction mixture. The reaction was monitored by mass spectrometry. **TAT-GWWWG_ Pam_2CSK_4_MDP (4)** was purified by HPLC (Solvent A: 0.1 % TFA in water, solvent B: 0.1% TFA in acetonitrile. Gradient: t 0-20 min, ramp 10% B to 90% B) to give a yield of 30%. MALDI-TOF: m/z calc'd for $[M+H]^+$ 5490.14 observed $[M+H]^+$ 5490.94

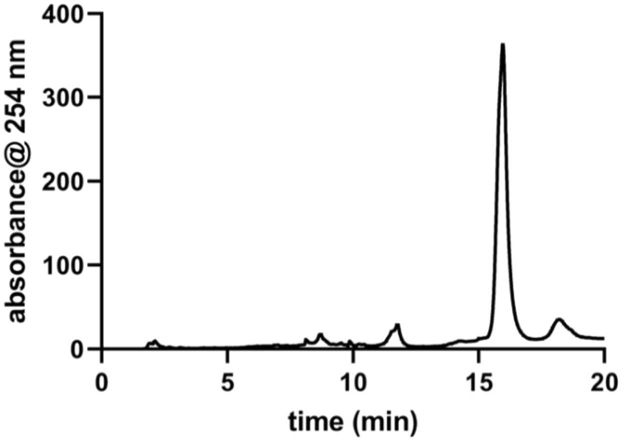

Figure S2: HPLC trace of **TAT-GWWWG_ Pam_2CSK_4_MDP** measured at 254 nm on a C8 analytical column. Solvent A: 0.1 % TFA in HPLC grade water, solvent B: 0.1% TFA in HPLC grade acetonitrile. Gradient: ramp 10% B to 90% over 20 min.

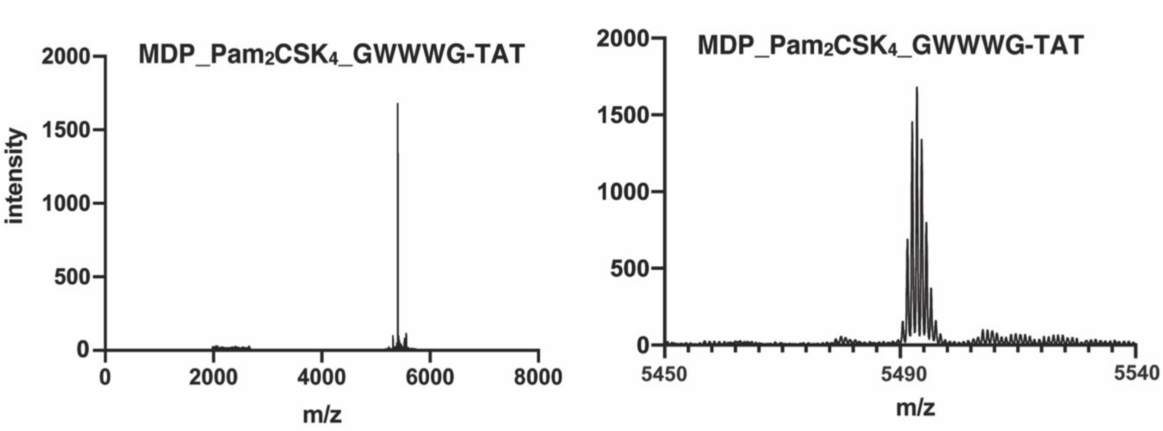

Figure S3: MALDI trace of **TAT-GWWWG_ Pam_2CSK_4_MDP** tri-agonist (left) and enlarged view of the major peak (right). Sample acquired in positive reflector mode using dihydroxybenzoic acid matrix.

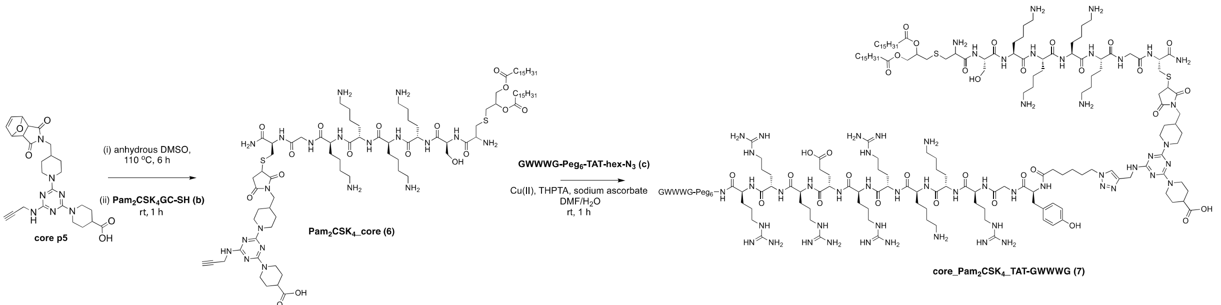

Scheme S4: Synthetic route to di-agonist **core_Pam$_2$CSK$_4$_TAT-GWWWG (7)**.

core_MDP_TAT-GWWWG (**8**): **core_MDP (2)** (5 mg, 0.035 mmol) was dissolved in DMF (200 μL). **GWWWG-Peg$_6$-TAT-hex-N$_3$ (c)** (10 mg, 0.004 mmol) dissolved in DMF (200 μL) were added to the solution. 200 μL of CuSO4•5H_2O (20 mM) solution and 400 μL of THPTA (50 mM) solution were mixed and added to the mixture. 200 μL of sodium ascorbate (100 mM) was then added to the reaction mixture. The reaction was monitored by mass spectrometry. **core_MDP_TAT-GWWWG (8)** was purified by HPLC (Solvent A: 0.1 % TFA in water, solvent B: 0.1% TFA in acetonitrile. Gradient: t 0-20 min, ramp 10% B to 90% B) to give a yield of 30%. MALDI-TOF: m/z calc'd for $[M+H]^+$ 4128.19, observed $[M+H]^+$ 4128.11

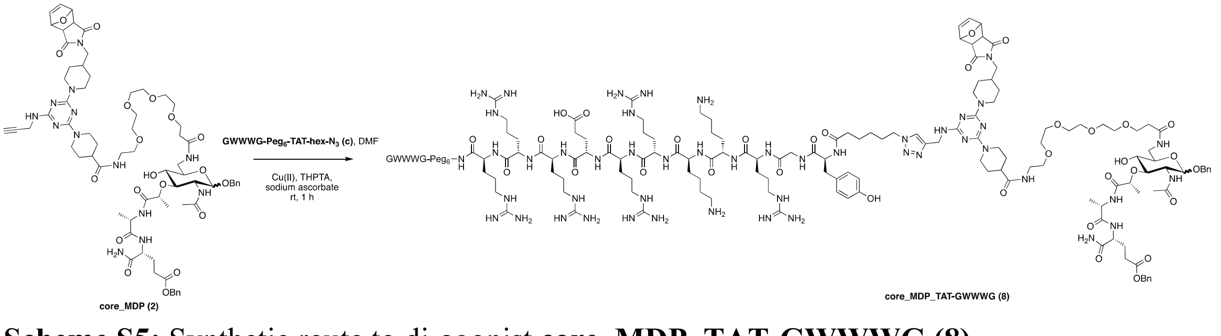

Scheme S5: Synthetic route to di-agonist **core_MDP_TAT-GWWWG (8)**.

Pam$_2$CSK$_4$_core (**6**)**:** Furan protected **core p5** (4mg, 0.007 mmol) was dissolved in anhydrous DMSO (3 mL) and stirred for 6 h at 110 °C to expose the maleimide. The reaction was monitored

by mass spectrometry. When furan was completely deprotected, the solution was cooled to room temperature. **Pam_2CSK_4GC (b)** (10 mg, 0.007 mmol) and DIPEA (500 uL) were added to the solution and stirred for 1 h. The reaction was monitored by mass spectrometry. **Pam_2CSK_4_core** (**6**) was purified by HPLC (Solvent A: 0.1 % TFA in water, solvent B: 0.1% TFA in acetonitrile. Gradient: t 0-20 min, ramp 10% B to 90% B) to give a yield of 82%. MALDI-TOF: m/z calc'd for [M+H] 1884.19, observed $[M+H]^+$ 1883.99.

core_Pam_2CSK_4_TAT-GWWWG (7): **Pam_2CSK_4_core** (**6**) (5 mg, 0.003 mmol) was dissolved in DMF (200 μL). **GWWWG-Peg_6-TAT-hex-N_3 (c)** (5 mg, 0.002 mmol) dissolved in DMF (200 μL) were added to the solution. 200 μL of CuSO4•$5H_2O$ (20 mM) solution and 400 μL of THPTA (50 mM) solution were mixed and added to the mixture. 200 μL of sodium ascorbate (100 mM) was then added to the reaction mixture. The reaction was monitored by mass spectrometry. **core_Pam_2CSK_4_TAT-GWWWG** (7) was purified by HPLC (Solvent A: 0.1 % TFA in water, solvent B: 0.1% TFA in acetonitrile. Gradient: t 0-20 min, ramp 10% B to 90% B) to give a yield of 30%. MALDI-TOF: m/z calc'd for $[M+H]^+$ 4589.69, observed $[M+H]^+$ 4590.10

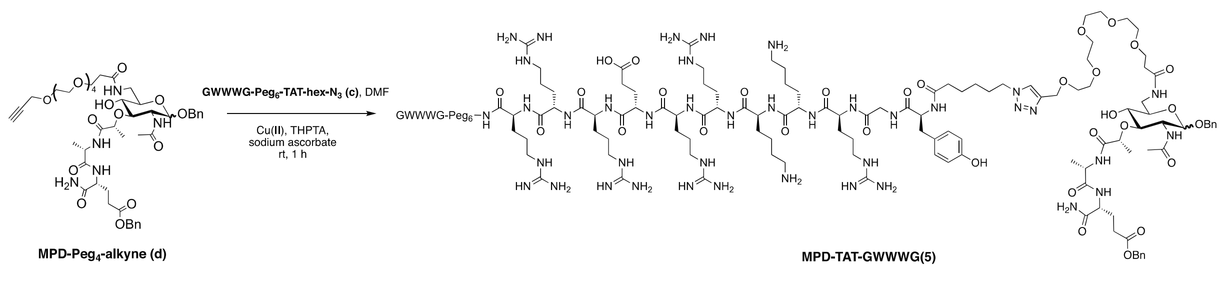

Scheme S6: Synthetic route to di-agonist **MDP_TAT-GWWWG (5)**.

MDP_TAT-GWWWG (**5**): **MDP-Peg_4-alkyne** (**d**) (5 mg, 0.005mmol) was dissolved in DMF (200 μL). **GWWWG-Peg_6-TAT-hex-N_3 (c)** (5 mg, 0.002 mmol) dissolved in DMF (200 μL) were

added to the solution. 200 μL of CuSO4•5H_2O (20 mM) solution and 400 μL of THPTA (50 mM) solution were mixed and added to the mixture. 200 μL of sodium ascorbate (100 mM) was then added to the reaction mixture. The reaction was monitored by mass spectrometry. **MDP_ TAT-GWWWG (5)** was purified by HPLC (Solvent A: 0.1 % TFA in water, solvent B: 0.1% TFA in acetonitrile. Gradient: t 0-20 min, ramp 10% B to 90% B) to give a yield of 30%. MALDI-TOF: m/z calc'd for $[M+H]^+$ 3663.96, observed $[M+H]^+$ 3664.16

^{1}HNMR and ^{13}CNMR spectra

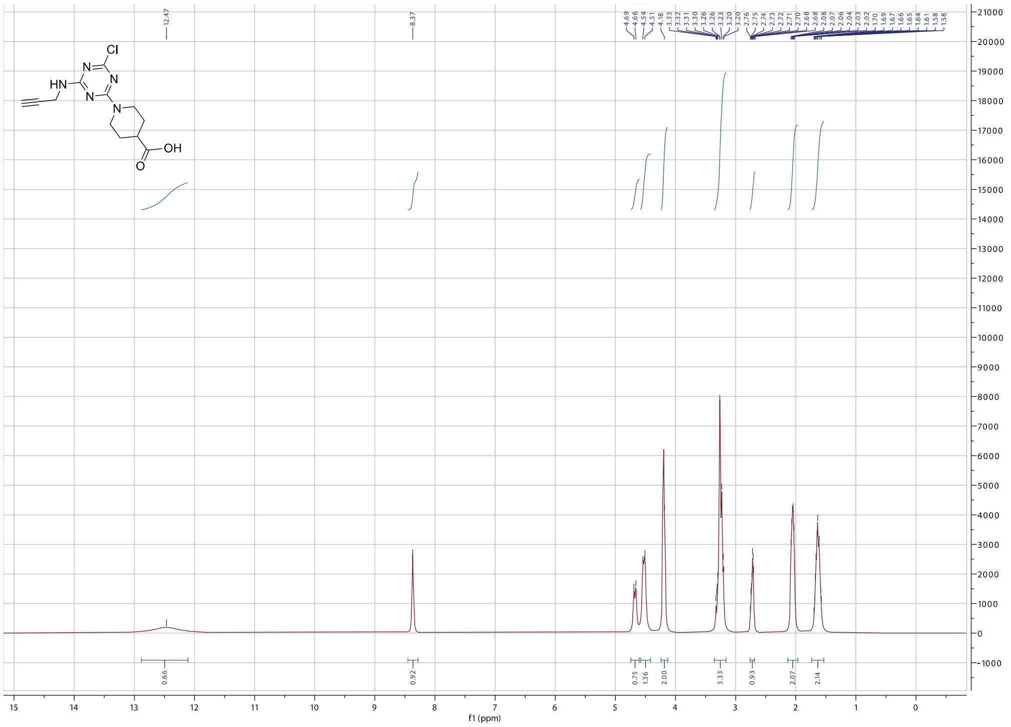

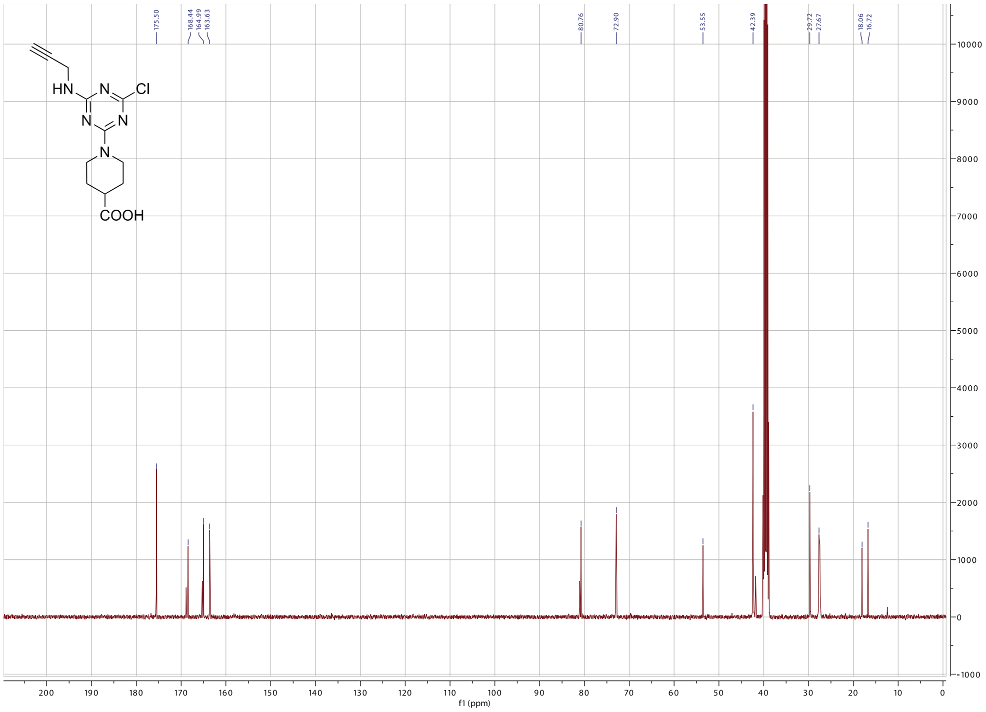

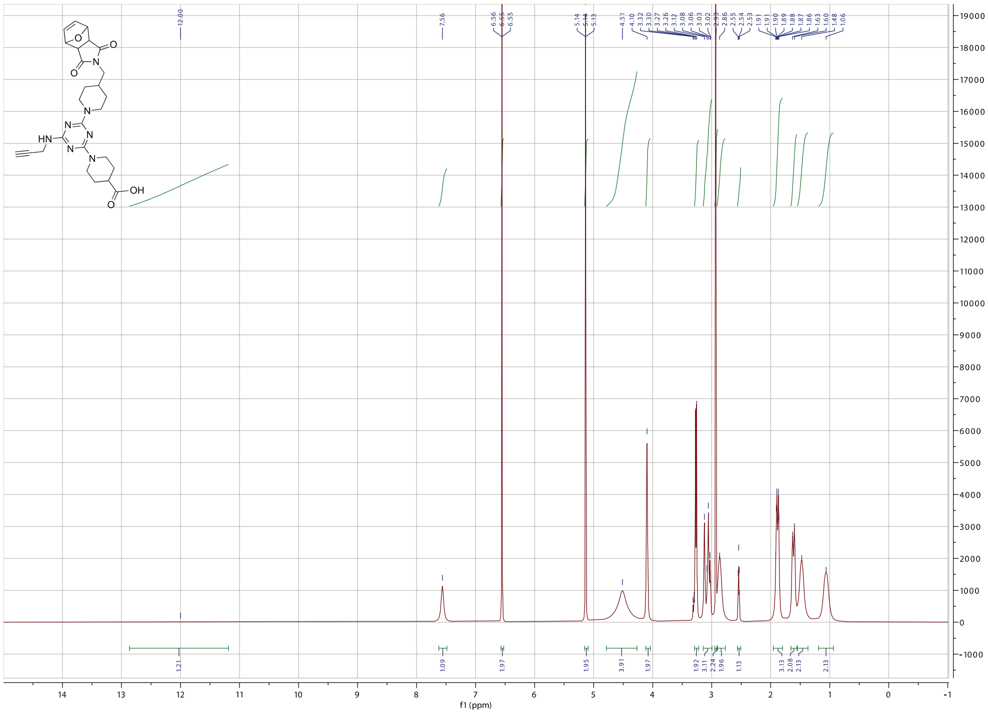

12.00
7.56
6.56
6.55
5.14
5.13
4.51
4.10
1.21
1.09
1.97
1.95
3.91
1.97
f1 (ppm)

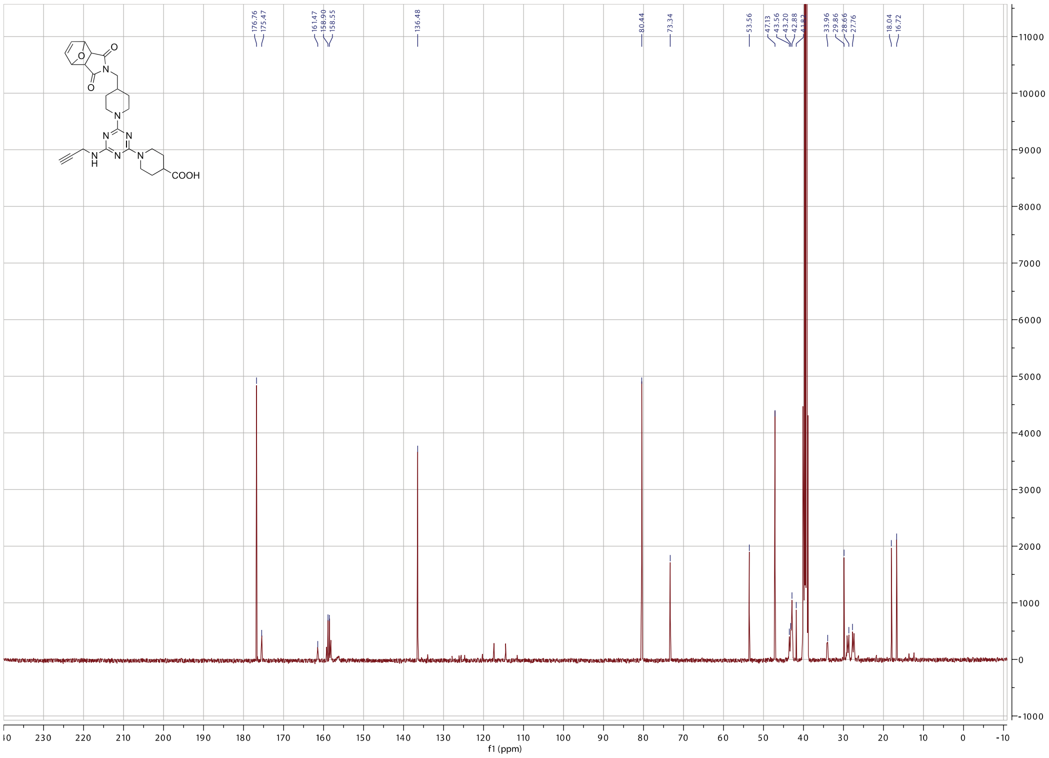

176.76
175.47
136.48
80.44
73.34
53.56
47.13
33.96
29.86
28.66
27.76
18.04
16.72
COOH
f1 (ppm)

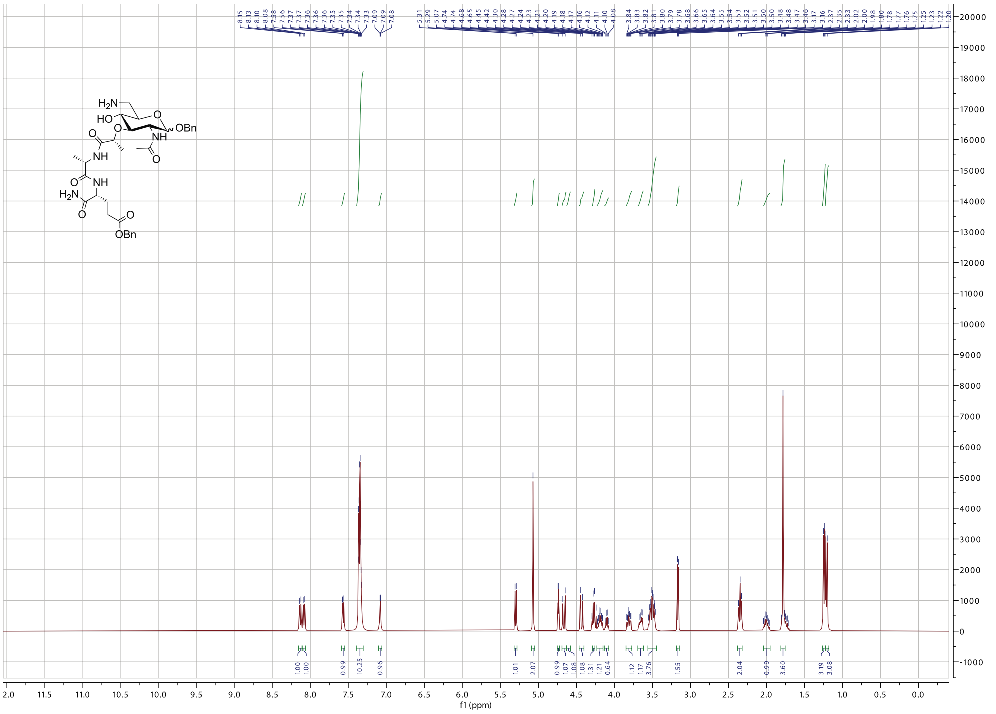
H2N
HO
OBn
NH
H2N
OBn
f1 (ppm)

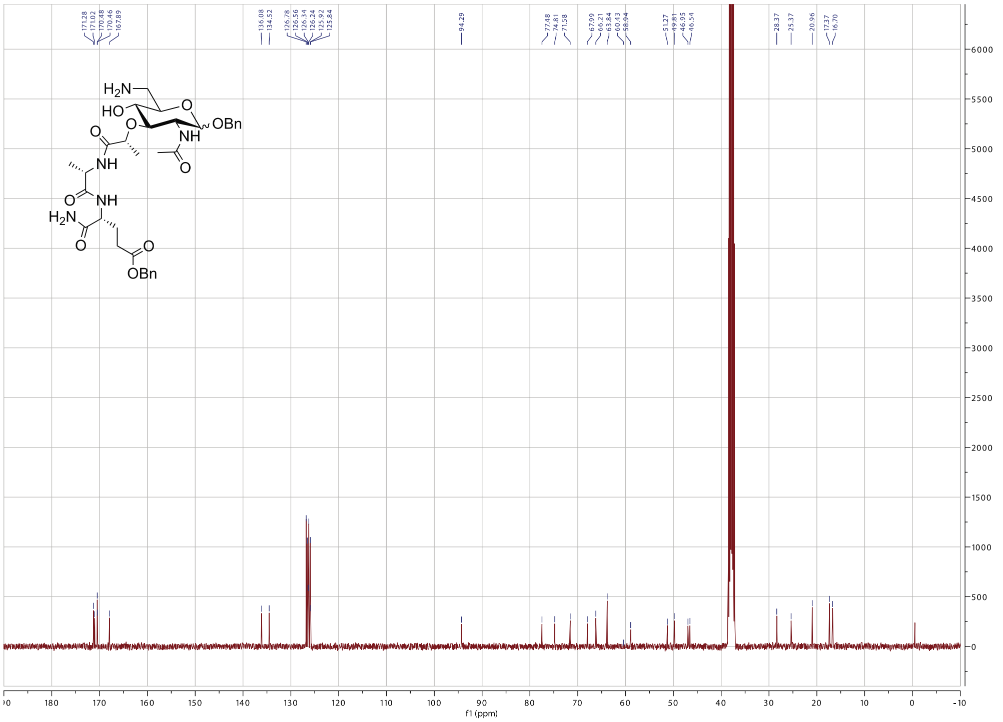
H2N
HO
OBn
NH
H2N
OBn
f1 (ppm)

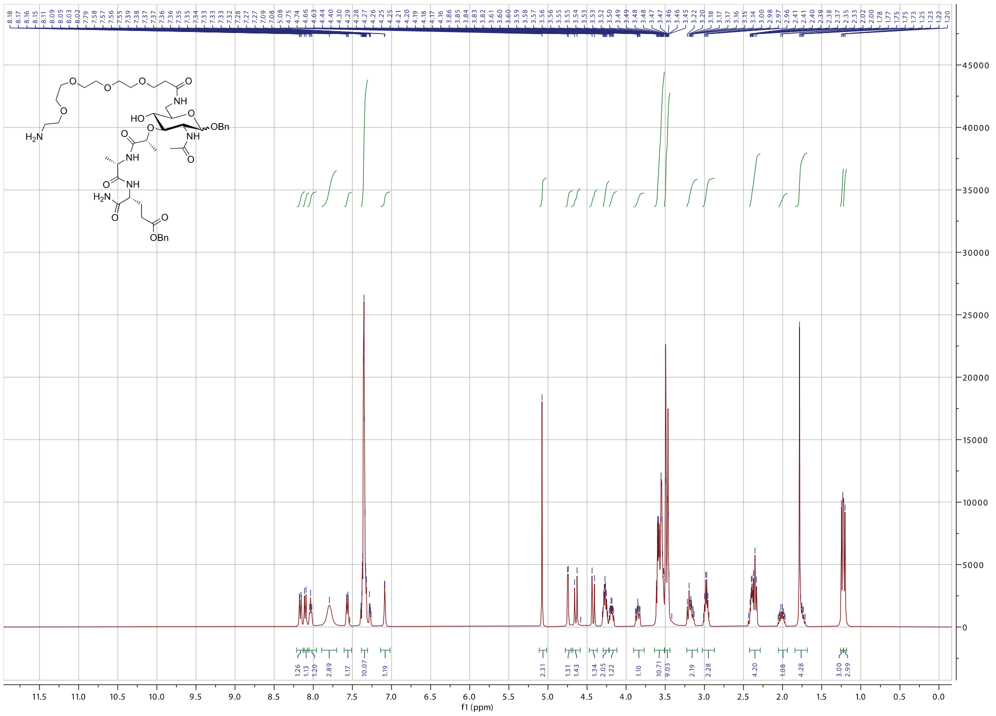

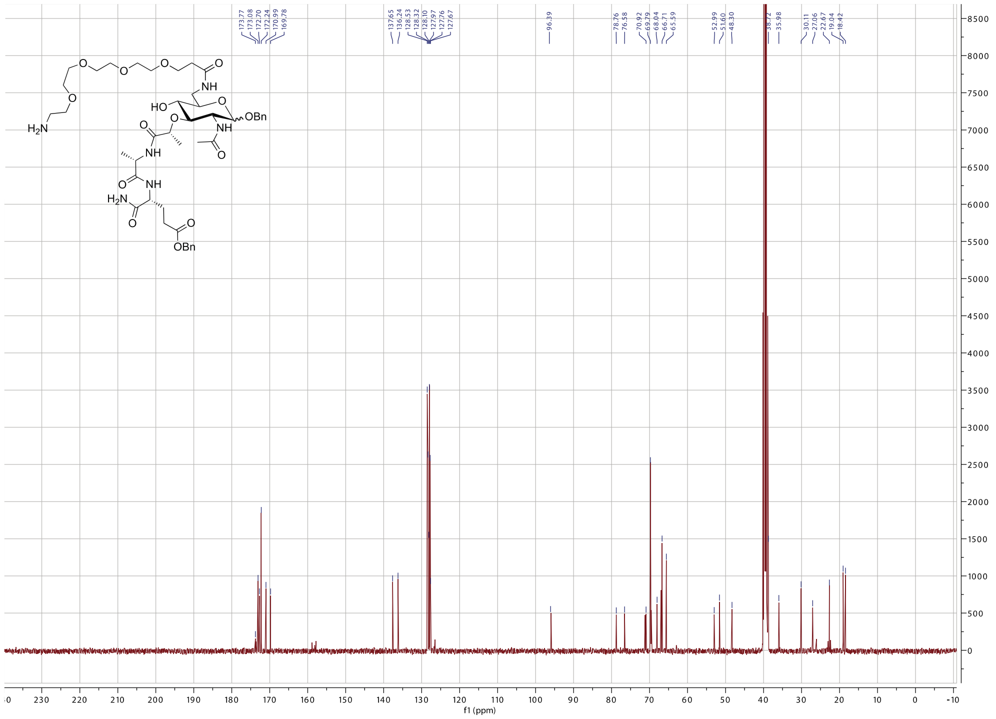

Figures from manuscript.

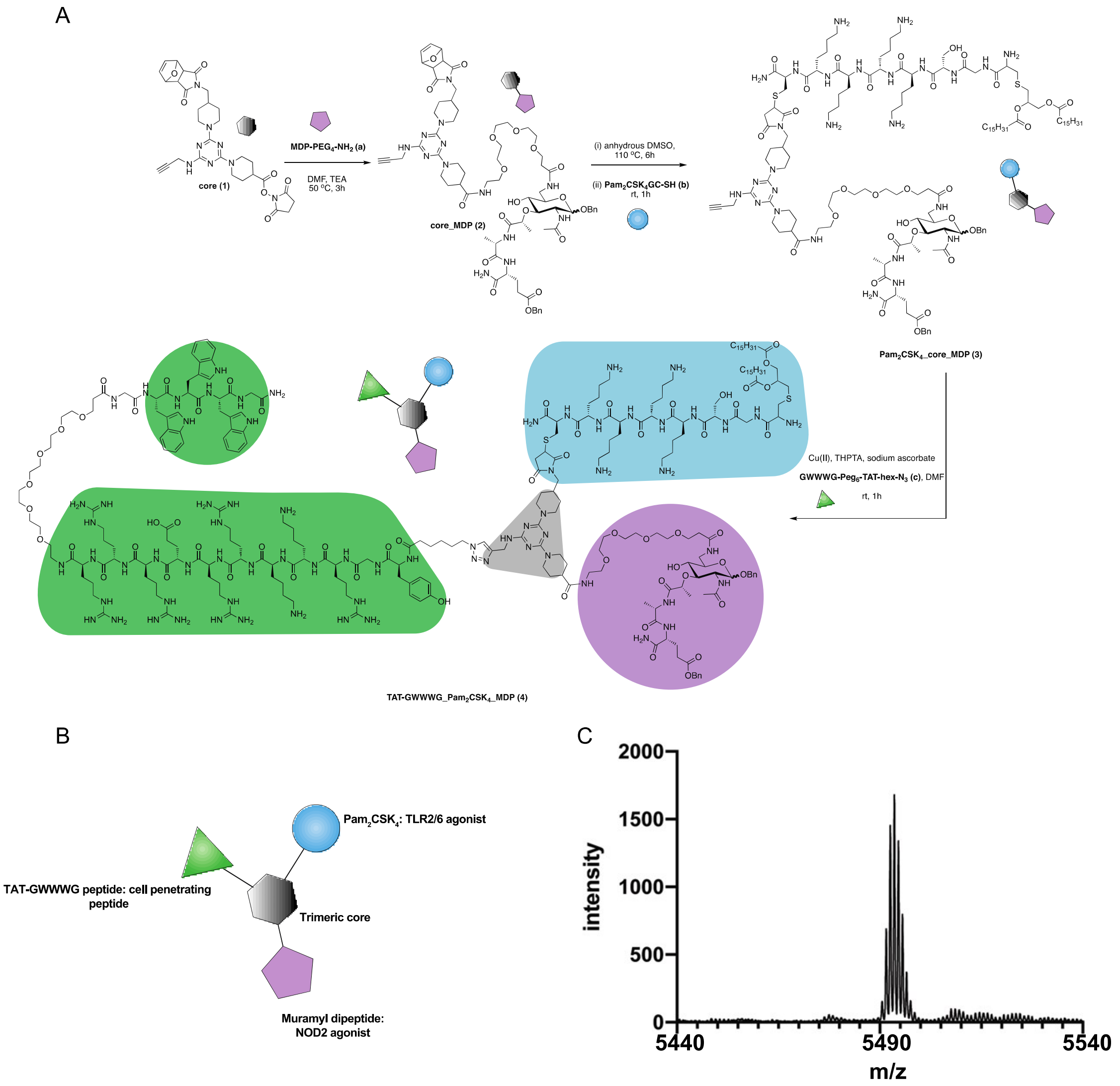

Figure 1. Synthesis of TAT-GWWWG_Pam2CSK4_MDP tri-agonist (4). (A) Synthesis Scheme (B): Schematic presentation of the linked tri-agonist. (C): MALDI trace of the linked tri-agonist.

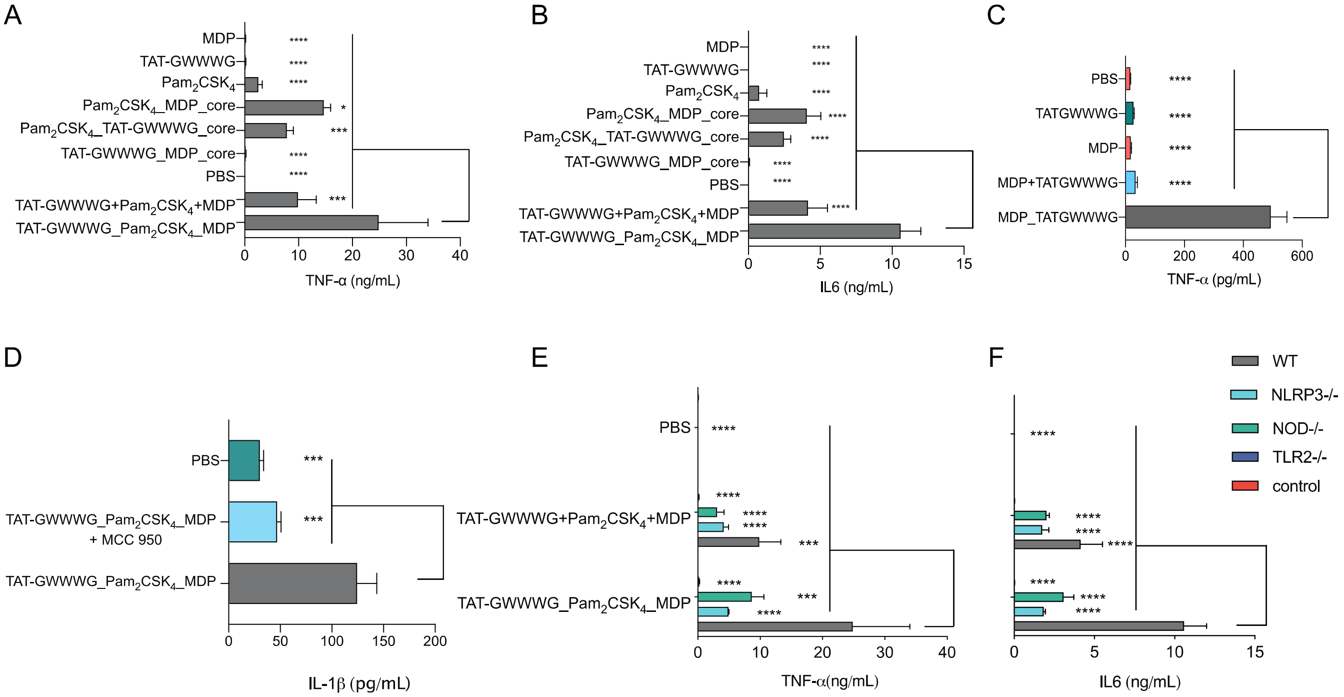

Figure 2. In vitro cytokine expression from BMDCs. (A), (B): Cells were incubated with linked PRR tri-agonist, a 1:1:1 (molar ratio) mixture of unlinked PRR agonists, various linked di-agonist combinations and single agonists (100 nM each) at 37°C for 6 h. TNF-α (A) and IL-6 (B) measured by CBA. (C): In vitro TNF-α expression from BMDCs as measured by ELISA. Cells were incubated with MPD_TATGWWWG (25 μM) or a 1:1 (molar ratio) mixture of the analogous unlinked agonists for 24 h at 37 °C. (D): Analysis of NLRP3 activity (IL-1β secretion) by ELISA. Cells were preincubated with NLRP3 inhibitor MCC-950 (10 mM) for 1 h and then stimulated with linked PRR tri-agonist (10 mM) at 37°C for 24 h. Inhibition of NLRP3 via MCC-950 results in loss of IL-1b activity. (E), (F): Analysis of TNF-α and IL-6 with WT, TLR2-/-, NOD2 -/- and NLRP3-/- cells treated with linked PRR tri-agonist or unlinked agonists (100 nM). Samples were run in triplicate, where * $p < 0.05$, *** $p < 0.001$, ****$p < 0.0001$. Statistical analysis performed using ANOVA with Turkey's multiple comparison test.

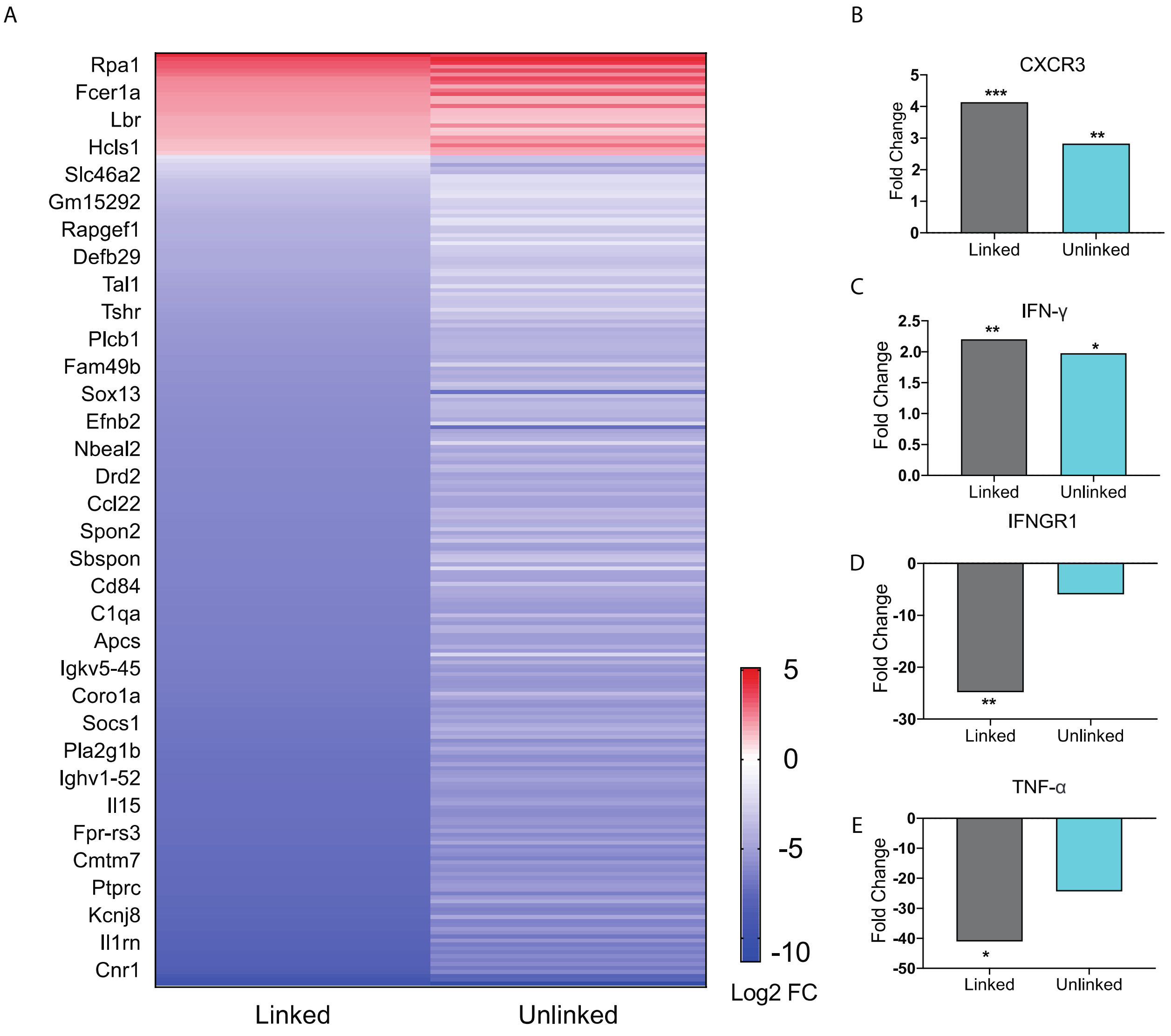

Figure 3. BMDC gene expression profile data. (A) Heat map of immune function related genes. Each figure represents the average of three independent experiments. BMDCs were incubated as untreated, or with either the linked or unlinked tri-agonist combination for 6 h at 37 °C. RNA was then extracted and sequenced on a NextSeq550. The gene expression of the BMDCs in response to unlinked and linked tri-agonist stimulation was compared to unstimulated BMDCs to determine the differential gene expression profiles. Included in the heatmap are only immune-associated genes with p value < 0.05 for either the linked or unlinked tri-agonists relative to unstimulated BMDCs and a 2-fold change in expression. (B), (C), (D), (E): Fold change in gene expression for CXCR3, IFN-γ, TNF-α, IFNGR1 in BMDCs in response to linked and unlinked tri-agonist combination where *p < 0.05, **p < 0.01, and ***p<0.001. P-values are calculated relative to unstimulated BMDCs.

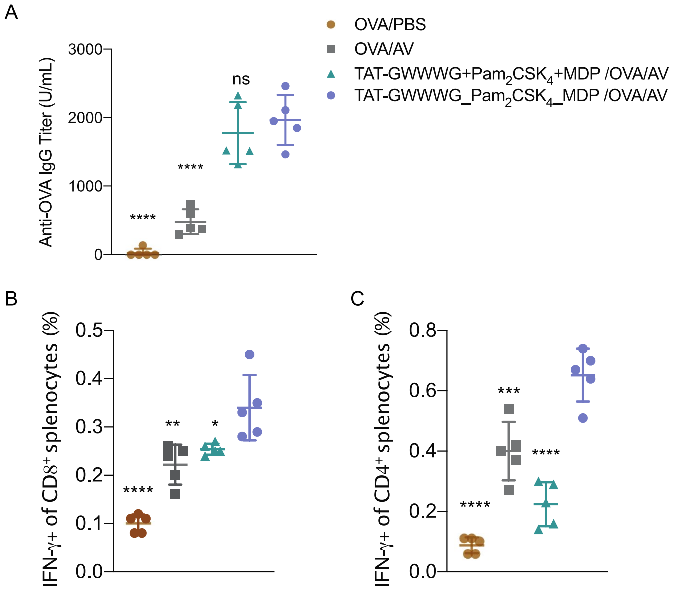

Figure 4. *In-vivo* Vaccination studies. Mice (n=5) were vaccinated on day 0 with OVA (100 mg) adjuvanted with PBS (vehicle control), or Addavax (25 mL), or 5 nmole each of unconjugated multi-PRR agonist, in Addavax (AV, 25 mL), or 5 nmole of linked tri-agonist in Addavax (25 mL). Final volume of each formulation was made 50 mL with PBS. Mice were given a vaccine boost on day 14. On day 24, sera, spleens were collected from mice. Antibody titer was measured by ELISA and T cell response was measured by intracellular cytokine staining. ns = non-significant, * $p < 0.05$, ** $p < 0.01$, *** $p < 0.001$, **** $p < 0.0001$. Statistical analysis is performed between the linked tri-agonist and indicated groups using ANOVA by the Turkey's multiple comparison test.

References

1 R. Banerjee, N. J. Pace, D. R. Brown, E. Weerapana, *J. Am. Chem. Soc.*, 2013, **135**, 2497–2500.

2 C. L. Grimes, D. K. Podolsky, E. K. O'Shea, *Bioorg. Med. Chem. Lett.*, 2010, **20**, 6061–6063.

3 T. J. Albin, J. K. Tom, S. Manna, A. P. Gilkes, S. A. Stetkevich, B. B. Katz, M. Supnet, J. Felgner, A. Jain, R. Nakajima, A. Jasinskas, A. Zlotnik, E. Pearlman, D. H. Davies, P. L. Felgner, A. M. Burkhardt, A. P. Esser-Kahn, *ACS Cent. Sci.*, 2019, **5**, 1137–1145.
